# Transfusion and Treatment of severe anaemia in African children (TRACT): a study protocol for a randomised controlled trial

**DOI:** 10.1186/s13063-015-1112-4

**Published:** 2015-12-29

**Authors:** Ayub Mpoya, Sarah Kiguli, Peter Olupot-Olupot, Robert O. Opoka, Charles Engoru, Macpherson Mallewa, Yami Chimalizeni, Neil Kennedy, Dorothy Kyeyune, Benjamin Wabwire, Bridon M’baya, Imelda Bates, Britta Urban, Michael Boele von Hensbroek, Robert Heyderman, Margaret J. Thomason, Sophie Uyoga, Thomas N. Williams, Diana M. Gibb, Elizabeth C. George, A. Sarah Walker, Kathryn Maitland

**Affiliations:** KEMRI-Wellcome Trust Research Programme, PO Box 230, Kilifi, Kenya; Department of Paediatrics, Mulago Hospital, Makerere University, PO Box 7072, Kampala, Uganda; Department of Paediatrics, Mbale Regional Referral Hospital Pallisa Road Zone, PO Box 921, Mbale, Uganda; Department of Paediatrics, Soroti Regional Referral Hospital, PO Box 289, Soroti, Uganda; College of Medicine, Department of Paediatrics and Child Health, University of Malawi, P/Bag 360 Chichiri, Blantyre 3, Malawi; Uganda Blood Transfusion Service, PO Box 1772, Kampala, Uganda; Mbale Regional Blood Bank, PO Box 284, Mbale, Uganda; Malawi Blood Transfusion Service, PO Box 2681, Blantyre, Malawi; Malawi-Liverpool-Wellcome Trust Clinical Research Programme, PO Box 30096, Chichiri, Blantyre 3, Malawi; Liverpool School of Tropical Medicine, Pembroke Place, Liverpool, L3 5QA UK; Global Child Health Group, Emma Children’s Hospital Academic Medical Centre, P.O. Box 22660, 1100 DD Amsterdam, The Netherlands; Division of Infection and Immunity, University College London, Cruciform Building, Gower Street, London, WC1E 6BT UK; MRC Clinical Trials Unit at University College London, Aviation House, 125 Kingsway, London, WC2B 6NH UK; Wellcome Trust Centre for Clinical Tropical Medicine, Department of Paediatrics, Faculty of Medicine, St Marys Campus, Norfolk Place, Imperial College, London, W2 1PG UK

**Keywords:** Children, Africa, Anaemia, Malaria, Sepsis, Transfusion, Micronutrients, Emergency medicine, Haemoglobinopathies, Antibiotic prophylaxis

## Abstract

**Background:**

In sub-Saharan Africa, where infectious diseases and nutritional deficiencies are common, severe anaemia is a common cause of paediatric hospital admission, yet the evidence to support current treatment recommendations is limited. To avert overuse of blood products, the World Health Organisation advocates a conservative transfusion policy and recommends iron, folate and anti-helminthics at discharge. Outcomes are unsatisfactory with high rates of in-hospital mortality (9–10 %), 6-month mortality and relapse (6 %). A definitive trial to establish best transfusion and treatment strategies to prevent both early and delayed mortality and relapse is warranted.

**Methods/Design:**

TRACT is a multicentre randomised controlled trial of 3954 children aged 2 months to 12 years admitted to hospital with severe anaemia (haemoglobin < 6 g/dl). Children will be enrolled over 2 years in 4 centres in Uganda and Malawi and followed for 6 months. The trial will simultaneously evaluate (in a factorial trial with a 3 x 2 x 2 design) 3 ways to reduce short-term and longer-term mortality and morbidity following admission to hospital with severe anaemia in African children.

The trial will compare: (i) R1: liberal transfusion (30 ml/kg whole blood) versus conservative transfusion (20 ml/kg) versus no transfusion (control). The control is only for children with uncomplicated severe anaemia (haemoglobin 4–6 g/dl); (ii) R2: post-discharge multi-vitamin multi-mineral supplementation (including folate and iron) versus routine care (folate and iron) for 3 months; (iii) R3: post-discharge cotrimoxazole prophylaxis for 3 months versus no prophylaxis. All randomisations are open. Enrolment to the trial started September 2014 and is currently ongoing. Primary outcome is cumulative mortality to 4 weeks for the transfusion strategy comparisons, and to 6 months for the nutritional support/antibiotic prophylaxis comparisons. Secondary outcomes include mortality, morbidity (haematological correction, nutritional and infectious), safety and cost-effectiveness.

**Discussion:**

If confirmed by the trial, a cheap and widely available ‘bundle’ of effective interventions, directed at immediate and downstream consequences of severe anaemia, could lead to substantial reductions in mortality in a substantial number of African children hospitalised with severe anaemia every year, if widely implemented.

**Trial registration:**

Current Controlled Trials ISRCTN84086586, Approved 11 February 2013

## Background

In sub-Saharan Africa severe anaemia (SA) in children is a leading cause of hospital admission, a major cause of direct mortality [1] and a key factor in the approximately 600,000 malaria deaths/year [2]. Guidelines developed by the World Health Organisation (WHO) encourage the rational use of blood transfusion to preserve this scarce resource and to reduce the risk of transfusion-transmitted infections [3]. However, the evidence base supporting these guidelines is weak, adherence is poor and national transfusion recommendations vary between sub-Saharan Africa countries [4]. Outcomes following SA are unsatisfactory with high rates of in-hospital (9–10 %) [5] and 6-month (12 %) mortality, and relapse or re-hospitalisation (6 %) [6], indicating that the current recommendations and their implementation are not working in practice. Further, the aetiology of SA is frequently multi-factorial, including potentially treatable co-morbidities such as bacteraemia and multiple vitamin deficiencies – key determinants of outcome [7] that are not addressed in current treatment guidelines. Although the 2 most recent systematic reviews (both published in 2000) indicated the need for formal evaluation of the restrictive transfusion policy supported by the World Health Organisation (WHO) in a controlled trial [5, 8], little progress has been made in the intervening decade. The poor outcomes and recurrent morbidity of children with SA warrant a definitive trial to establish best transfusion and treatment strategies to prevent both early and delayed mortality and relapse.

### Current WHO recommendations

#### Transfusion

To avert overuse of blood products the WHO advocates a conservative transfusion policy, reserving blood for children with a haemoglobin (Hb) < 4 g/dl (or < 6 g/dl if accompanied by complications). Although not systematically evaluated, this conservative transfusion policy has been incorporated in WHO paediatric hospital practice guidelines. However, the specific recommendations in these guidelines contain inconsistencies and ambiguities [9] resulting in variation in practice across African countries, most particularly in the subgroup with ‘uncomplicated’ SA (Hb 4–6 g/dl without severe symptoms) where transfusion avoidance is recommended [4, 8]. A Cochrane review including the only 2 African randomised controlled trials (RCTs) [10, 11] conducted to date (involving 114 and 116 children randomised to blood transfusion or oral haematinics) concluded that there was insufficient information on whether routinely giving blood to clinically stable children with SA either reduces death or results in a higher haematocrit measured at 1 month, and indicated the need for a definitive trial [8].

Overall mortality in children with Hb < 4 g/dl or SA with life-threatening complications is 15 % [5]. Clinical studies in Kenya [12, 13] have shown that profound anaemia (Hb < 4 g/dl) is independently associated with death (odds ratio; OR = 2 · 5), as is SA (defined in this study as a Hb < 5 g/dl) complicated by reduced consciousness (OR = 7 · 4) or respiratory distress (OR = 4 · 1). Many deaths occur within 48 hours of admission, with 25–50 % [14, 15] occurring within 6 hours. In the FEAST trial, which enrolled children with shock, a higher case fatality was found in those with anaemia compared to those without anaemia, irrespective of intervention group [16]. In children with uncomplicated SA – Hb 4–6 g/dl without prostration or respiratory distress – overall case fatality is 4–6 %, being lower in parasitaemic children (2–3 %) [17] than in those with negative malarial slides (8–10 %) [12]. The ratio of complicated to uncomplicated SA is commonly 1:1 [18].

Current transfusion guidelines are conservative not only in terms of criteria applied for administering a transfusion at all, but also in terms of the volume of blood transfused. Currently, 20 ml/kg of whole blood (or 10 ml/kg packed cells) are recommended for all levels of anaemia below Hb < 6 g/dl [19]. Using standard formulae to calculate volume required [20] this under-treats children with profound anaemia by approximately 30 % and this volume may not, therefore, be sufficient to correct anaemia [4].

### Other treatment recommendations for severe anaemia

WHO treatment guidelines deal specifically with acute treatment of malaria and with folate and iron deficiency, together widely held as the most important causes of anaemia. There are no specific recommendations for subsequent infection prophylaxis (including against malaria) [3]. In the only comprehensive case-control study (SeVana) of children hospitalised with SA in Africa [7], key aetiological factors for SA were bacteraemia (OR = 5.3; 95 % confidence interval; CI 2.6–10.9), malaria (2.3; 1.6–3.3), hookworm (4.8; 2.0–11.8), HIV infection (2.0; 1.0–3.8), vitamin A deficiency (2.8; 1.3–5.8) and vitamin B_12_ deficiency (2.2; 1.4–3.6). A subsequent publication, reporting the long-term outcome of children in this study, HIV infection was found to be the major risk factor both for 18-month post-discharge mortality (hazard ratio (HR) 10.5, 95 % CI 4.0–27.2) and for recurrence of SA (HR 5.6, 95 % CI 1.6–20.1). Children admitted with bacteraemia were also at an increased risk of post-discharge all cause mortality (HR 2.2, 95 % CI 0.8–5.6).

With respect to current treatment recommendations neither iron nor folate deficiencies were factors for SA being less prevalent among cases than controls (without SA) in the SeVana study. Thus, although folate supplementation is recommended, folate deficiency was not found in the Malawian SeVana study [6], in agreement with previous reports [21] and observations that folate supplementation in anaemic children with malaria failed to raise Hb concentrations [22]. Moreover, vitamin B_12_ and vitamin A supplementation are not recommended in guidelines for the management of SA. Iron supplementation is effective for reduction of iron deficiency and anaemia in iron-deficient children. However, a community-based randomised controlled trial in Zanzibar designed to evaluate the impact of zinc and iron plus folic acid supplementation on morbidity and mortality in young children showed that supplementation may also be associated with adverse effects, specifically increased risk of hospitalisation (primarily due to malaria and infectious disease), and mortality in malaria-endemic areas [23]. The WHO has revised its recommendations to advise that iron and folic acid should only be targeted towards those who are anaemic and at risk of iron deficiency. Establishing iron status in children hospitalised with SA, and more generally in paediatric populations living in malarial areas, is technically challenging [24, 25] and is rarely available in resource-limited hospitals, making the implementation of WHO guidelines challenging in the very areas that are most affected. The development of micronutrient powders (eg Nutromix™ or Sprinkles™; Hexagon Nutrition Pvt Ltd, Nashik, Maharastra, India), as a novel approach for delivering iron and other micronutrients, offers a chance to correct relevant nutrient deficiencies [26] and provide iron in lower doses; with good adherence in population-based studies [27].

With regard to infection prophylaxis the substantial mortality benefits (allied with extremely low rates of toxicity) associated with cotrimoxazole prophylaxis in HIV-infected children [28] have generally been attributed to reductions in bacterial infections [29, 30]. Of note, these benefits have been observed even in areas of high background resistance [31]. The fact that mortality benefits cannot be attributed solely to pneumonia [29, 30] raises the intriguing possibility that cotrimoxazole may act on a number of different pathways – the most important with regards to SA relapse being enteropathy and intestinal permeability, although any benefits of cotrimoxazole on microbial translocation and/or systemic immune activation, or on reducing recurrent infections during recovery from SA, could also impact longer-term morbidity. Cotrimoxazole has been shown to be effective in preventing malaria in HIV-uninfected children aged > 5 years [32], and in HIV-exposed uninfected (HIV-uninfected children born to HIV-infected mothers) and HIV-infected children [33], despite high levels of background parasite resistance to sulphamethoxazole.

In summary, the best available evidence suggests that key factors for poor long-term outcome following SA hospitalisation are nutritional factors and recurrent bacterial infection, the strongest potentially modifiable underlying causes of morbidity and mortality which we propose to address in this trial.

## Methods/Design

### Study objectives

The primary objective of the trial is to identify effective, safe and acceptable interventions to reduce short-term and longer-term mortality and morbidity following admission to hospital with SA in sub-Saharan Africa. There are 2 hypotheses being tested:

1. A liberal rather than a conservative blood transfusion policy will decrease mortality (cumulative to 4 weeks) in children admitted to hospital with SA (Hb < 6 g/dl).
2. Supplementary multi-vitamin multi-mineral (MVMM) treatments or additional cotrimoxazole prophylaxis or both for 3 months post discharge will reduce rates of readmission, SA relapse, re-transfusion or death (cumulative to 6 months) compared to current recommendations (iron and folate) and anti-helminthics in all (anti-helminthics if aged > 1 year).

### Secondary objectives

i. To identify the most cost-effective interventions to reduce early mortality, and assess their budget impact
ii. To determine efficacy of long-term support strategies (MVMM and cotrimoxazole prophylaxis) on other markers of nutritional status and causes of death
iii. To determine the effect of transfusion strategies and long-term support strategies on markers of inflammation and immunological activation and function
iv. To identify the mechanism of action of the most effective interventions through focussed investigations of human genetic polymorphisms, molecular diagnostics, immunological activation, markers of gut barrier dysfunction, and haematological and nutritional response

### Study design and population

TRACT is a multicentre randomised controlled trial of 3954 children aged 2 months to 12 years admitted to hospital with a Hb < 6 g/dl. Children will be enrolled over 2 years from 2 countries and followed for 6 months. The trial will simultaneously evaluate 3 ways to reduce short-term and longer-term mortality (primary endpoint) and morbidity following admission to hospital with SA in sub-Saharan Africa using a 3 x 2 x 2 factorial design. All randomisations will be open. Inclusion/exclusion criteria are detailed in Table 1.

**Table 1:** Inclusion and exclusion criteria

Inclusion criteria	Exclusion criteria
Aged 2 months to 12 years	Malignancy or other terminal illness
Severe anaemia (Hb < 6 g/dl) on the day of admission to hospital	Acute trauma or burns as main reason for admission
Care-giver willing/able to provide consent	Surgery as main reason for admission
	Chronic renal or liver failure
	Signs of bi-ventricular heart failure
	Known congenital or valvular heart disease (non-surgically corrected)
	Children who are exclusively breast fed (thus unable to take nutritional support)

### Trial interventions

**Fig. 1:**
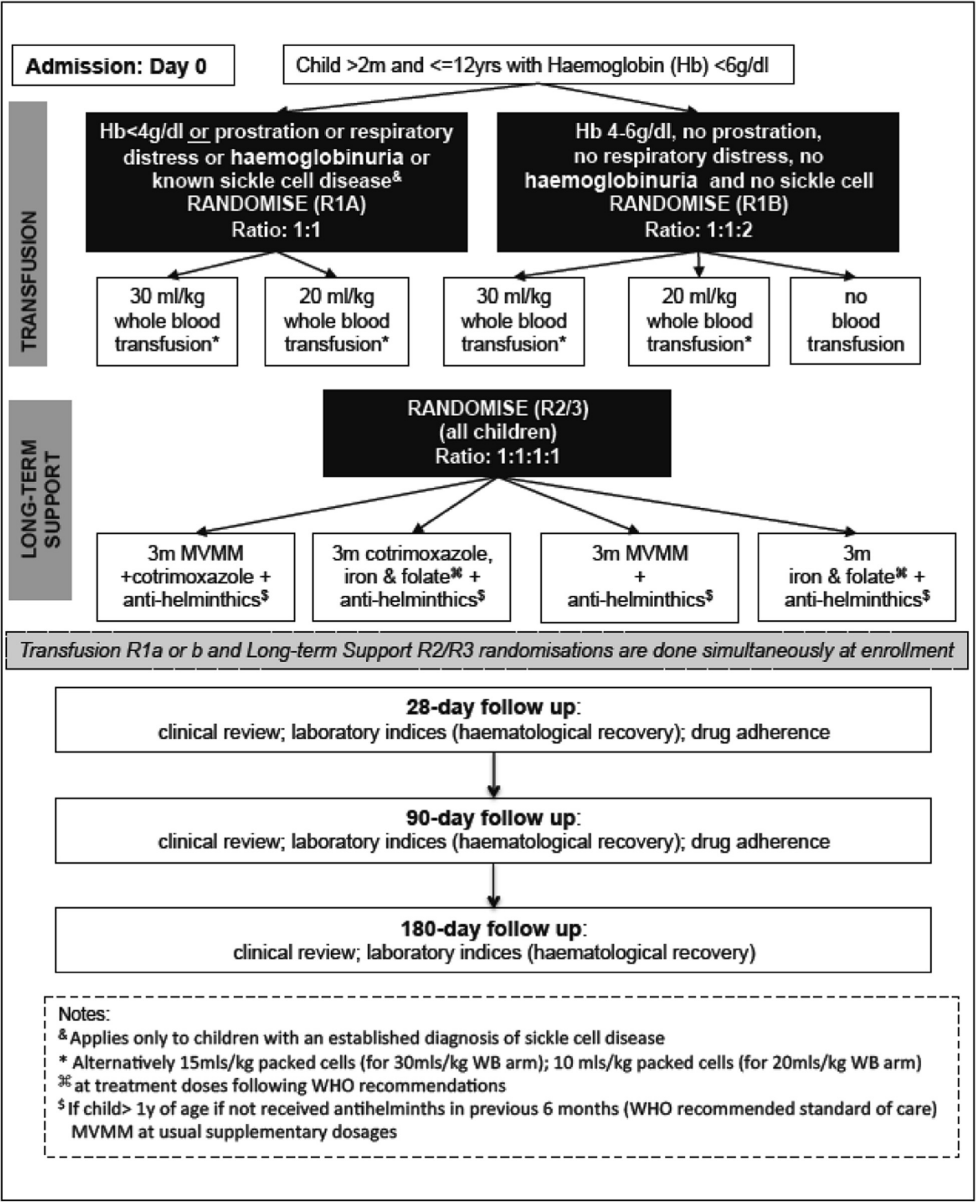
Trial scheme for the Transfusion and Treatment of severe anaemia in African children trial (TRACT)

Each intervention addresses one of the potential approaches to reducing mortality and morbidity in children with SA (Fig. 1: Trial flow schema)

- R1: Immediate liberal transfusion (30 ml/kg) versus immediate conservative transfusion (20 ml/kg) versus no transfusion (the last strategy only for children with uncomplicated SA with Hb 4–6 g/dl).
- R2: Post-discharge MVMM supplementation for 3 months (which includes folate and iron) and anti-helminthics if aged > 1 years versus routine care (folate and iron at standard treatment doses (varies with age) for 3 months) and anti-helminthics if aged > 1 year.
- R3: Post-discharge cotrimoxazole prophylaxis for 3 months versus no prophylaxis.

R1 addresses both conservative aspects of current guidelines: ‘whether to give blood’ in uncomplicated SA (4-6 g/dl without complications), and ‘how much blood to give’ in all children with SA. The transfusion and post-discharge interventions will be open-label for reasons of practicality and compliance.

#### Potential for interactions between the trial interventions

Because the transfusion, nutritional and antibiotic prophylaxis interventions approach different mechanisms for reducing short-term and long-term mortality/morbidity following SA, we consider that important interactions between randomised groups are unlikely. Further, any interactions that do exist are likely to be quantitative (slightly smaller/larger effects) rather than qualitative (effect on one background, no effect on another).

### Ethics approvals

The trial protocol was reviewed and obtained approval from the Imperial College Research Ethics Committee (ICREC_13_1_11). In Uganda the trial was approved by the Research Ethics Committee in Uganda, Makerere University School of Medicine Research and Ethics Committee, Kampala (#REC ref 2013–050) and in Malawi by the College of Medicine Research and Ethics Committee, (P.03/13/1365). In both countries regulatory authorities’ approval was also received.

### Consent

Prospective written, informed consent will be obtained from parents or guardians of children who are considered to be sufficiently stable. Parents or guardians will be given an information sheet in their usual language containing details of the TRACT trial. The sheet will be read aloud to those who are unable to read. Parents and guardians will be encouraged to ask questions about the trial prior to signing the consent form. The right of the participant to refuse to participate without giving reasons must be respected. However, we anticipate a number of children presenting as emergencies where delay in study enrolment, and thus treatment, through a consent procedure would be unacceptable. A two-stage consent process in this circumstance and the details are covered under section ‘Consent process for severe life-threatening anaemia’ below.

### Treatments given in the trial

#### Standard management

All trial patients will receive standard of care (SOC) including antibiotics (given intravenously or orally) and/or anti-malarial drugs following national guidelines, based on WHO syndromic patient management [3]. We will collect data on all administered drugs. Antipyretics, anticonvulsants and treatment for hypoglycaemia will be administered according to nationally agreed protocols. If required, maintenance fluids will be run at 3–4 ml/kg/hour irrespective of age until the child can drink and retain oral fluids. At discharge from hospital all aged children > 1 year will be receive empiric treatment for helminths (500 mg mebendazole or 400 mg albendazole in Malawi) in accordance to current recommendations (SOC) regardless of randomised allocation.

### Trial treatments

#### Randomisation procedures

Randomisation in each part of the factorial will be stratified by centre and the other randomisations in the factorial. Randomisation lists, using variable block sizes, will be generated and kept at the Medical Research Council Clinical Trials Unit (MRC CTU), London. The randomisation envelopes and clinical packs will be prepared before the trial, with 1 set for complicated SA (R1a) and 1 for uncomplicated SA (R1b). Eligible children will be screened and recruited at the time of hospital admission. At enrolment sealed consecutively numbered opaque envelopes (opened in strict numeric order) will assign a TRACT trial number and indicate a clinical pack number. The clinical pack will be within the first 10 packs in the study filing cabinet but will not necessarily be the first remaining clinical pack number (an additional level of allocation concealment). Once opened, the clinical pack contains case report forms (CRFs) and a card which confirms the TRACT trial number, and simultaneously assigns transfusion interventions R1a/b (according to SA strata), micronutrient support (R2) and antimicrobial prophylaxis (R3) randomly. A similar system has worked well in the emergency care trial, FEAST [16].

The randomisation procedures and adherence to allocated treatments are reviewed at each independent monitoring visit. The process for this is detailed within the monitoring Standard Operating Procedure (SOP). The monitors are requested to check at each participating site whether: a) the randomisation envelopes are being correctly stored and used; b) whether enrolled children are allocated to the correct arm and c) that the allocated trial treatments (transfusion, MVMM/iron or folate and cotrimoxazole) were received by the child. It is also monitored electronically through central database monitoring, and also that both the Data Monitoring Committee (DMC) and the Trial Steering Committee (TSC) will monitor overall adherence to each of the randomised allocations.

#### R1 transfusion strategies

At enrolment children will be assessed at admission and will be divided into 2 groups for randomisation (R1a and R1b: see Table 2) based on: i) haemoglobin level, and ii) assessment of clinical severity children or complications (reduced conscious level; respiratory distress, acute history of haemoglobinuria or an established diagnosis of sickle cell disease).

**Table 2:** Transfusion sub-groups

R1a Complicated severe anaemia: Hb < 4 g/dl *or* a Hb < 6 g/dl *plus* 1 or more signs of severity or complications
R1b Uncomplicated severe anaemia: Hb ≥ 4 and < 6 g/dl without any of the severity features or complications

#### R1a complicated severe anaemia

Children fulfilling these severity criteria will be randomly allocated on a 1:1 basis to receive one of the following:

- ○ Whole blood transfusion 20 ml/kg, alternatively 10 ml/kg packed cells; or
- ○ Whole blood transfusion 30 ml/kg, alternatively 15 ml/kg packed cells

#### R1b uncomplicated severe anaemia

Children with an Hb 4–6 g/dl without any severity features will be randomly allocated on a 1:1:2 basis to receive one of the following:

- ○ Whole blood transfusion 20 ml/kg alternatively 10 ml/kg packed cells, or
- ○ Whole blood transfusion 30 ml/kg alternatively 15 ml/kg packed cells, or
- ○ No transfusion (control, SOC)

### Transfusion treatment schedule

A clinician or medical officer will prescribe the blood, using a calculator, to determine the volume of whole blood required (20 or 30 ml/kg). If only packed cells are available then the clinician must re-calculate the equivalent volumes of packed cells (10 or 15 ml/kg). Transfusions will be administered in gauged blood burettes; an initial aliquot (2 ml) will run into a sterile apex tube using an aseptic technique (and ensuring that the tip of the infusion set does not touch anything, to prevent contamination) and 1 drop taken from this to record the Hb and haematocrit of the donor blood. Whole blood will be run over 3–4 hours and packed cells can be administered over 2–3 hours.

For all children in the trial an additional, or initial (for SOC control group in R1b only), transfusion(s) will be permitted after 8 hours (at the point of the first reassessment of Hb) for children who still have either: (i) profound anaemia Hb < 4 g/dl, irrespective of other signs of severity; (ii) SA 4–6 g/dl and one or both de novo signs of severity (respiratory distress or impaired consciousness); (iii) uncorrected SA 4–6 g/dl in children with an acute history of haemoglobinuria or known sickle cell disease. Early sampling of Hb (<8 hours from baseline), and additional transfusion, will be permitted in children randomised to any group in the R1b strata (uncomplicated SA) developing de novo signs of severity.

If a child randomised to no-transfusion control (R1b only) meets the above criteria, they will receive 20 ml/kg whole blood or 10 ml/kg packed cells, as recommended by the WHO [3]. Children randomised to initially receive blood (R1a and R1b) who subsequently meet the above criteria will follow their randomisation arm: that is they will receive either an additional transfusion of 20 ml/kg or 30 ml/kg of whole blood (or 10 ml/kg or 15 ml/kg packed cells respectively). Any child who has already received 2 transfusions and subsequently fulfils the criteria above will receive a maximum of 20 ml/kg (or 10 ml/kg packed cells) irrespective of randomisation. Frusemide or other diuretics will be prescribed at the discretion of the attending physician and not used routinely in the trial.

#### R2 micronutrient support

Simultaneously to R1 randomisations, all children entering the trial will also be randomly allocated on a 1:1 basis to receive either multi-vitamin multi-mineral mix (MVMM: Nutromix™ which contains iron, folate and other MVMM) or iron and folate alone (at WHO-recommended doses) for 3 months post discharge. Nutromix™ has been specifically designed for children of 6–24 months of age with SA [34–36]. The formulation, meets the recommended nutrient intake (RNI), particularly for vulnerable groups during emergencies [37]. RNI is defined as the daily dietary intake of a nutrient sufficient to meet the nutrient requirements of nearly all apparently healthy individuals in a specific population group, usually by age and sex. The dosage is 1 sachet to be taken daily by the child [26] and will be prescribed at the time of discharge from hospital (or 5 days from randomisation in those not discharged by this timepoint). In those children receiving iron (syrup or tablets) and folate tablets, for children aged < 2 years the recommended dosages are 25 mg iron: 100–400 μg folate; and for children > 2 years and < 12 years, 60 mg iron: 400 μg folate. Iron, folate and MVMM will all be given for 3 months, according to WHO guidelines for the management of SA.

The use of nutritional supplementation, including MVMM randomisation, will be pragmatic in that all children for whom these supplements should be received, according to WHO or national guidelines (e.g. those initially admitted with severe malnutrition), will receive them. Children aged < 6 months who are not weaned (fully breast-fed) will be excluded from the trial. For children with severe malnutrition, iron-containing supplements are not recommended during the first 7 days of acute rehabilitation (WHO guidelines) [3] but can be used effectively after this. For children with severe malnutrition discharged on ready-to-use therapeutic food (RUTF) which contains MVMM, children will essentially ignore their allocated MVMM randomisation, but will receive their standard post-discharge supplementation within the RUTF which would be recorded on study CRFs. The number of children with severe malnutrition as their admission diagnosis is expected to be small (< 5 %).

#### R3 antimicrobial prophylaxis

Children will be randomly allocated on a 1:1 basis to receive either receive: (i) cotrimoxazole prophylaxis for 3 months post discharge or (ii) no antibiotic prophylaxis post discharge (control, SOC). Cotrimoxazole dispersible tablets (240 mg: trimethoprim 40 mg/sulphamethoxazole 200 mg) will be used and dosing will follow WHO recommendations for prophylaxis in HIV-infected children: age 2 to 6 months: 120 mg; age 6 months to 5 years: 240 mg; children > 5 years: 480 mg [38]. The dispersible tablets may be taken with water or mixed with feeds. Cotrimoxazole will be prescribed from discharge (or 5 days from randomisation in those not discharged by this timepoint). The cotrimoxazole prophylaxis randomisation will be pragmatic in that all children for whom cotrimoxazole prophylaxis should be prescribed according to WHO or national guidelines (e.g. HIV-infected children) will receive it regardless of randomisation, and no child in whom it is contraindicated (e.g. known GP6D deficiency according to local testing) will receive it. Such children will essentially ignore their allocated cotrimoxazole randomisation; any cotrimoxazole received per guidelines would be recorded on study CRFs. The number of children with these conditions is expected to be small (< 5 %). HIV-infected children will receive antiretrovirals and will continue in the trial with HIV management and follow-up tailored in collaboration with local HIV clinics.

### Co-enrolment guidelines

Patients will not ordinarily be permitted to participate in any other clinical intervention trial or research protocol while on the TRACT trial. Participation in other studies that do not involve an intervention may be acceptable, following agreement from the TRACT trial management group (TMG). The TRACT TMG will consider co-enrolment of TRACT participants onto other trials where the interventions do not conflict with the TRACT objectives on a case-by-case basis.

### Measurement of endpoints

#### Assessment during hospital

The clinicians will complete a detailed clinical review on the CRF and perform a physical examination at enrolment. A symptom checklist and targeted physical examination will be performed at each subsequent clinical assessment. Children will be intensively monitored on the day of admission by the clinical team, and during any transfusion and then reviewed daily by the study team until discharge, with Hb testing performed at least 8 hourly in the first 24 hours, and daily thereafter. At each review conscious level, vital signs (heart rate, oxygen saturation, respiratory rate, axillary temperature, blood pressure) will be recorded, and examinations will specifically review the child for solicited adverse events. The doctor will be responsible for documenting and reporting serious adverse events (SAEs). Admission and final diagnoses will be recorded in the CRF.

### Follow-up

A symptom checklist and targeted physical examination will be performed at each clinic visit post discharge. Medical history since last visit including hospital re-admissions, transfusions, specific solicited adverse events, and grade 3 or 4 adverse events related to nutritional and antibiotic interventions will be documented by a doctor, including severity and likely relationship of any adverse events to trial interventions. At Day 28 adherence to and acceptability of MVMM and/or cotrimoxazole will be queried by carer self-report, and carers will be provided with a supply of drugs sufficient to last for the next 2 months (Day 90 since admission). Blood and other tests and sample storage will require a maximum of 4 mls heparinised blood (plasma) and 1 ml into ethylenediaminetetraacetic acid (EDTA) (for pathogen diagnostics). At Day 90, adherence/acceptability will again be recorded, but no more supplements/antibiotics will be given. Any participants requiring further care at their Day 180 visit will transferred into the routine clinics at the centre where the trial is being conducted. Locator maps and contact numbers will be obtained to facilitate follow-up.

### Withdrawing from the trial and protocol treatment discontinuation

In consenting to the trial, parents or guardians and children are consenting to trial treatment, data collection and follow-up. If a patient chooses to discontinue any part of their trial treatment, they should always be encouraged not leave the whole trial and to return for follow-up, providing they are willing. After the participant has entered the trial the clinician remains free to give alternative treatment to that specified in the protocol at any stage if he/she feels it is in the participant’s best interest, but the reasons for doing so should be recorded. In these cases the participants remain within the study for the purposes of follow-up and data analysis. All carers and participants are free to withdraw at any time from the protocol treatment without giving reasons and without prejudicing further treatment. If they do not wish to remain on trial follow-up, however, their decision will be respected and the patient will be withdrawn from the trial completely.

Withdrawal from the transfusion intervention or control arms is unlikely, given that most transfusions are given within the first 12 hours of admission. Severe allergic reaction (toxicity) or Transfusion Related Acute Lung Injury (TRALI) is included as a secondary endpoint and is relevant only to children receiving transfusion. It will not be a reason to withdraw the child from the trial, but further transfusions should be withheld. The child should continue with their cotrimoxazole/MVMM allocation wherever possible.

### Outcomes

Primary outcome and secondary outcomes are detailed in Table 3. The methods for assessing efficacy include:

**Table 3:** Outcome measures

Primary outcome
Cumulative mortality to 28 days for the transfusion strategy comparison, and to 180 days for the nutritional support/antibiotic prophylaxis comparison
Secondary outcomes
Mortality: at 48 hours, 28 days, 90 days and 180 days (cumulative) where not the primary outcome
Morbidity: endpoints relating to the specific mechanisms of action of each intervention:
re-admission to hospital
haematological:
proportion achieving correction of anaemia (defined by the WHO as Hb > 9 g/dl) at 48 hours, 28 days, 90 days and 180 days
development of new profound anaemia (Hb < 4 g/dl) during acute admission or development of severe anaemia (Hb < 6 g/dl) post discharge
nutritional: changes in weight and mid-upper arm circumference (MUAC) at 90 days and 180 days
anti-infection: changes in inflammatory markers (C-reactive protein (CRP) , procalcitonin), incidence of bacterial infections and malaria at 28 days, 90 days and 180 days
solicited adverse events: suspected transfusion reactions: febrile reactions, TRALI (Transfusion Related Acute Lung Injury) (any grade); grade 3–4 toxicity of cotrimoxazole, MVMM or standard iron/folate
serious adverse events
costs and cost-effectiveness

#### Clinical events

Survival status will be recorded at discharge and each subsequent visit (28 days, 90 days and 180 days following admission). Any patient lost to follow-up before 6 months without withdrawing consent will be traced for vital status. Other SAEs will be reported as and when the doctor becomes aware of them. The details reported will include bedside observations, laboratory data, and additional clinical narrative. During the index admission, any child fulfilling criteria for a new or additional transfusion will be recorded. At all subsequent visits hospital admissions and requirement for transfusion will be solicited.

#### Haematological recovery

Haemoglobin will be recorded at 8-hour periods up to 24 hours, then daily until discharge, then at 28 days, 90 days and 180 days.

#### Safety and adverse event reporting

The symptom checklist used at each visit will explicitly prompt for symptoms relating to possible drug toxicities. Additional safety blood tests or investigations may be performed to investigate symptoms or monitor emergent laboratory test abnormalities as clinically indicated. Serious, solicited and grade 3 or 4 adverse events will be reported on the CRF. Adverse events (clinical and laboratory) will be graded according to toxicity/severity grading.

#### Adherence

Adherence to nutritional and prophylaxis drugs will be assessed in all participants at each visit by pill counts for tablets, and nurse-administered questionnaire to the child’s carer, and where appropriate to the child (at the discretion of the nurse or doctor depending on age).

#### Health economics

The trial will measure healthcare-related costs in trial participants, starting at randomisation and continuing for the duration of follow-up. Information on hospitalisations (number, reason, and duration of stay) and other healthcare resource utilisation (visits to healthcare centres, medications) will be collected by carer interview at the 28-day, 90-day and 180-day visits. The economic evaluation will be conducted from the health services perspective. Unit costs will be attached to resource use, using the best available estimates of long-run marginal opportunity cost, to obtain a cost per patient over the period of follow-up. Routinely available national unit costs will be used where possible with local estimations where necessary. There will also be a budget impact analyses of the consequences of adopting the interventions on the health sector budgets, in each of the countries of the trial.

#### Anti-infective

Changes in inflammatory markers (eg CRP) (all participants) will be measured retrospectively together with the incidence of bacterial infections, acute febrile illness and malaria at 28 days, 90 days and 180 days from carer interview, blood cultures and non-culture-based molecular diagnostics on stored samples.

### Methods of reducing bias

Protection against bias is principally provided by a completely objective primary endpoint (mortality). Any child lost to follow-up before 6 months will be traced for vital status. Cause of death (and other clinical secondary endpoints) will be adjudicated by an Endpoint Review Committee (ERC), blinded to randomised allocations. The ERC will adjudicate on causes of death and whether fatal and non-fatal SAEs were unlikely, possibly/probably or uncertainly related to each of the randomised interventions (transfusion volume, MVMM, cotrimoxazole), if the child were to be receiving them (without knowledge of actual randomisation), to protect from bias in this open trial. The randomisations to transfusion strategies and long-term support are open because of logistical constraints – it is impossible to blind transfusion or nutritional strategies since MVMM is in a sachet and iron and folate are largely provided separately as a syrup and crushable tablets or tablets alone. Whilst cotrimoxazole prophylaxis could be blinded, the intention of this pragmatic strategy trial is to also assess likely impacts of non-compliance to taking cotrimoxazole on an antibiotic prophylaxis strategy in SA.

### Sample size calculation and statistical analysis

#### Sample size

The sample size calculation is based on the following assumptions:

- 80 % power, 2-sided alpha = 0.013 to allow for 4 comparisons (see below)
- SA cases are 50 % complicated (< 4 g/dl or 4–6 g/dl with prostration/respiratory distress/known sickle cell disease/haemoglobinuria) and 50 % uncomplicated (4–6 g/dl without prostration, respiratory distress, known sickle cell disease or haemoglobinuria) [12] (and Dr. Olupot-Olupot personal communication)
- Mortality (cumulative) at 48 hours and 4 weeks is 11 % and 16 % respectively in complicated SA, and 4 % and 9 % in uncomplicated SA
- The cumulative rate of re-admission, SA relapse and re-transfusion at 6 months is 12.5 % in both complicated and uncomplicated SA (in addition to mortality above)
- For the primary comparison of transfusion versus no transfusion in uncomplicated SA at 4 weeks, the minimum clinically relevant difference is a 50 % relative reduction (R1b): for the other primary comparison of transfusion volume (20 versus 30 ml/kg) at 4 weeks (R1a and 1b), the minimum clinically relevant difference is a 30 % relative reduction. The minimum clinically relevant difference is larger for the transfusion versus no transfusion question as provision of safe blood at all will require greater resources than provision of slightly larger versus slightly smaller blood volumes. As the same relative difference translates to a far larger absolute difference at higher event rates, for the primary comparison at 6 months (R2/3) the minimum clinically relevant difference is a 5 % absolute reduction (see below for control group event rates). Then:
  - (R1b) comparison of transfusion versus no transfusion (50 % reduction from 9 % control mortality at 4 weeks) requires 1460 uncomplicated SA cases (1:1 allocation to 30/20 ml/kg:no transfusion, 730 in no transfusion group, 365 receiving 20 ml/kg and 365 receiving 30 ml/kg transfusions)
  - (R1a and 1b) if the overall ratio of uncomplicated:complicated SAs is 1:1 (ie 50 % of each type), then within the subgroup randomised 1:1 to 30 versus 20 ml/kg, the ratio will be 1:2 because this comparison excludes the 50 % of uncomplicated SA randomised to no transfusion. Overall mortality at 4 weeks in this group will, therefore, be 13.67 % (0.33 × 9 % + 0.67 × 16 %). The comparison of 30 versus 20 ml/kg (30 % reduction, to 9.57 %) requires 2798 SAs, 1399 per group (split 466 uncomplicated, 933 complicated)
  - Therefore, comparing required sample sizes for R1a and R1b, to address both parts of the transfusion question (R1) we need slightly more uncomplicated SA children per group from (R1a and 1b – *n* = 466) than (R1b – *n* = 365), and therefore the total sample size is 3730 cases (933 × 2 = 1866 complicated, 466 × 4 = 1864 uncomplicated)
  - (R2/3) the comparison of multi-vitamins versus SOC (1:1) and cotrimoxazole prophylaxis versus SOC (1:1) requires 3162 SA cases, assuming 50 % are complicated and 50 % uncomplicated, to detect a 5 % absolute reduction from average control mortality of 25 % (0.5 × 21.5 % + 0.5 × 28.5 % at 4 weeks).

Thus, a sample size of 3730 SA cases would allow the multiple comparisons above to be made. Assuming a 6 % loss to follow-up by 6 months increases this to 3954 SA cases. As the effect sizes are reasonably large on the relative scale (> 30 % reduction), inflation factors which adjust for the factorial design are close to 1. However, assumptions are more sensitive to the relative contribution of uncomplicated:complicated SA. Capping the uncomplicated SA strata at 2000 cases (ie recruiting at least 1950 complicated SA) retains at least 80 % power to detect the differences above independently of variations in the contributing proportions of complicated SA.

Randomising uncomplicated SA (R1b) 1:1:2 between 30 ml/kg:20 ml/kg:no transfusion provides greater power for the comparison of no transfusion (SOC) versus transfusion in this group because the final randomisation ratio for transfusion:no transfusion is 1:1. In contrast, a 1:1:1 randomisation in this strata would produce a 2:1 transfusion:no transfusion ratio which has lower power.

### Statistical analysis

The analyses will be described in detail in a full Statistical Analysis Plan. In brief, each intervention is hypothesised to be superior to SOC and, therefore, the proposed analysis is intention-to-treat, including all randomised patients. The primary analysis will compare: a) transfusion versus no transfusion (strata b only) and b) 20 ml/kg versus 30 ml/kg (strata a and b) in terms of the proportion of children with fatal outcome 28 days after randomisation. Primary outcome analysis will use time-to-event methods (Kaplan-Meier, log-rank test, proportional hazards models) to the timepoints specified for primary and secondary outcomes, stratified by centre and anaemia severity at baseline. Correction of anaemia will also be analysed using time-to-event methods.

Pre-specified subgroup analyses will include each of the other randomised allocations (ie exploration of interactions in the factorial design), together with the other randomisation stratification factor (centre) and the anaemia stratification factor (a versus b) for the transfusion randomisation. We will also investigate a priori whether there was any evidence for a different impact of the interventions according to the following categorical variables: previous receipt of a transfusion (ever or at another health centre in this illness); speed (rate) at which the transfusion is administered; fever; malaria; microbiological evidence of sepsis (blood culture or retrospective molecular diagnosis); HIV; known or previously undiagnosed sickle cell disease.

For the cotrimoxazole prophylaxis and MVMM supplementation randomisations the primary analysis will be intention-to-treat based on all randomised participants, as above. However, secondary analyses will be restricted to: (i) patients discharged alive, and (ii) patients discharged alive in whom these interventions were neither mandated nor contraindicated (ie excluding HIV-infected children and those with known GP6D deficiency from the cotrimoxazole randomisation, and excluding children admitted for severe acute malnutrition from the supplementation randomisation).

Secondary outcome measures will be analysed using time-to-event methods or normal linear regression for continuous variables. The frequency of hospital re-admissions and adverse events will be tabulated by body systems and by randomised groups, and the number of events experienced by each participant will be compared across randomised groups using Fisher’s exact test.

For the within-trial cost-effectiveness analysis, the differential cost of the treatment interventions will be related to their differential outcomes in terms of the primary outcome. The relative cost-effectiveness of the alternative forms of management will then be assessed using standard decision rules and a full stochastic analysis will be undertaken. A cost-utility analysis will also be conducted using a standard approach. The within-trial analysis will be augmented by extrapolation beyond the trial follow-up using decision-analytic modelling. The aim of this analysis will be to predict the implications of any difference in clinical endpoints in the trial for subsequent quality-adjusted survival duration and long-term resource costs. This will inform the question of whether any differences in drug costs between the treatment groups are offset by reduction in other treatment costs or health improvements in the long term.

### Safety and ethical issues

#### Consent process for severe life-threatening anaemia

A number of children will present as emergencies where delay in study enrolment, and thus treatment, through a consent procedure would be unacceptable. For the FEAST trial we developed and received ethical approval to use a two-stage consent process in this circumstance [39] and subsequently evaluated the views and experiences of key stakeholders (parents, nurses and clinicians) [40]. Verbal assent will be sought from parents or guardians by the admitting medical team, if it is considered that the full consent process would significantly delay treatment allocation, and consequently could be detrimental to the child’s health. Full consent will be sought once the child’s clinical condition has been stabilised. Caregivers will be provided with a brief verbal description of the trial and will be given the opportunity to ‘opt out’ of clinical research. The clinician will later sign the verbal assent form, which will be filed with the consent form. However, if a child is enrolled on verbal assent by the guardian or guardian and dies before informed consent is obtained, the local and Imperial College Research Ethics Committee have approved that informed consent will not be obtained in these cases but that their data will be included in the overall analysis.

### Treatment strategies

Owing to high demand for blood for transfusion and limited resources each day the clinical teams will check with the transfusion services the quantities of blood available for transfusion. Screening and enrolment will not occur if there are no current supplies of blood and when stocks are limited and screening and enrolment will only be conducted for children without severe complicated anaemia (eligible for R1a strategies) since this group would ordinarily receive blood transfusion. Children with uncomplicated anaemia (eligible for R1b strategies) will only be enrolled on days where blood transfusion supplies are judged to be adequate. The numbers of patients enrolled each day will vary from site to site and depend upon blood supplies and requirement by non-study children.

Both MVMM and cotrimoxazole prophylaxis have been widely used in children with minimal risk. Although substantial efforts have been made to ensure the safety of blood, failure to correctly cross-match and/or infected blood have the potential to cause harm. The trial will directly evaluate whether these potential risks are outweighed by improved survival. TRACT teams are working closely with the local blood transfusion services (BTS) to ensure that recommended safety and quality control practices are being maintained.

A pilot study conducted in 2 sites in Uganda in 160 children fulfilling TRACT eligibility criteria evaluated the safety of the higher volume of whole blood transfusion proposed (30 ml/kg) compared to the standard volume (20 ml/kg) – ClinicalTrials.Gov NCT01461590. The trial study was able to demonstrate safety, and qualitative data on feasibility and operational components of implementation of the study protocol, adherence to volumes of transfusion and efficacy [41].

The trial will be recruiting patients with severe and complicated anaemia with a high mortality. At the start of the trial all sites will receive emergency care training, including triage of those at highest risk. All patients will be closely monitored so that clinical deteriorations can be identified at the earliest opportunity and appropriate therapy initiated. In general the trial sites in Africa have considerable experience with this population and this will serve to minimise the risks to the patients and the trial. A detailed risk assessment will be conducted prior to starting the trial.

The principles of the International Conference on Harmonisation Good Clinical Practice (ICH GCP) guidelines require that both investigators and sponsors follow specific procedures when notifying and reporting adverse events or reactions in clinical trials. Severe adverse event management follows a dedicated SOP developed for the previous FEAST trial. Blood samples are required from all study children. However, the volumes of blood required would be minimised wherever possible and be kept well within the maximum locally agreed volumes.

## Discussion

The operationalisation of a transfusion trial, investigating more liberal strategies in terms of which children with SA would benefit from a transfusion and how much to transfuse (in terms of volume), resulted in a number of logistic, scientific and ethical challenges. Whilst the demand for blood for transfusion is high in sub-Saharan Africa, with children under 5 years and women of reproductive age accounting for over 75 % of all transfusions, stocks of blood for transfusion remain limited. Although the data are very old and not comprehensive (from 2004) the WHO estimates that the minimum blood requirement for countries in sub-Saharan Africa to be 10 to 20 units per 1000 population per year; yet on average only 2.3 units of blood are donated per 1000 population [42, 43]. This poses a major threat to the operational feasibility of the trial reliant upon busy, over-burdened blood transfusion services that face acute shortages of blood when demand is high, especially during malaria seasons [44]. Added to this were concerns raised about the safety of using a larger volume of blood for transfusion as proposed in the trial.

In our justification for the trial, we distilled these concerns into three key elements. First, the safety aspects of increased transfusion volume. We also considered that this concern should extend to the number of repeat transfusions received during admission, rather than the just the volume of blood given per transfusion. Second, the potential that more liberal strategies may increase demand for blood and divert scarce resources and finally, the difficulties in providing accurate volumes of blood to ensure minimal wastage and reliable protocol adherence. At the design phase of the trial we noted that the published scientific literature on all these aspects was sparse. With regards to repeat transfusions, a sub-analysis of the FEAST trial recently published indicates that of 1422 children transfused, 322 (23 %) received 2 or more transfusions, the proportion being greater (212/612, 35 %) in those with hemoglobin < 4 g/dl at enrolment [4]. Thus, multiple transfusions, occurring in routine practice, not only incur additional resource utilisation of both transfusion supplies and healthcare manpower (across the whole chain of blood preparation, transport, clinical management and monitoring of the transfusion) but also expose children to the added risks of infection, transfusion reaction and adverse events. In addition, to test the safety of the higher volume proposed for initial transfusion we conducted a pilot Phase II trial (Tx30: ClinicalTrials.Gov: NCT01461590) [41]. The trial evaluated the safety and efficacy of a 30 ml/kg whole blood versus 20 ml/kg (SOC) in 160 children hospitalised with SA with respect to haematological recovery, adverse events and the need for additional transfusion. We demonstrated a superior outcome in children randomised to 30 ml/kg in terms of hemoglobin recovery at 24 hours (the primary outcome) and through to 28 days (global *p* < 0.0001); averting the requirement for repeat transfusion (5 % versus 15 %, *p* = 0.06) and with no indication that the higher initial volume resulted in an increase in adverse or fatal events compared to those in the 20 ml/kg arm. The trial also demonstrated that the use of digital scales to validate the accuracy of volumes of blood supplied by transfusion service, simple formulae and gauged blood burettes ensured reliable protocol adherence and decreased wastage. The initial volume actually infused followed randomisation strategy (within 5 ml/kg) in 80 (98 %) patients in the 20 ml/kg arm and 75 (96 %) in the 70 ml/kg arm [41]. We believe these additional data provide reassuring support for safety and feasibility of the TRACT trial.

To operationalise the trial on a day-to-day basis the manual of operations stipulates that the clinical teams must first check with the transfusion services that blood is available and the adequacy of stocks of blood packs. If there are no supplies then at these times no screening and enrolment to the trial will occur. If, however, supplies are at a critical level, children with severe and complicated anaemia, eligible for R1a randomisation, should be screened and enrolled since they would ordinarily receive a blood transfusion [3]. However, at times when blood supplies are critically low then children with SA (Hb < 6 g/dl) without complications (and thus eligible for R1b randomisation) will not be enrolled to ensure blood is not diverted for trial use.

One final issue pertinent to the transfusion strategies is the potential for substantial variability of the haematological quality of the donor whole blood and red cell concentrates. We anticipate that this would influence haematological recovery and, therefore, have put in place measures to quality control this aspect. For each pack of blood transfused in the trial, prior to the blood being run into the gauged transfusion burette, the nurse or doctor will gently agitate the blood pack to ensure the blood is thoroughly mixed and there is no settling of red cells. Following this, the initial blood volume that will run down the administration line from the gauged burette will have an aliquot run into a sterile apex tube using an aseptic technique (to prevent contamination) for Hb and haematocrit testing of the donor blood.

### Trial status

Enrolment to the trial started September 2014 and is currently ongoing. The 1000^th^ patient was recruited on 7^th^ June 2015.

## Abbreviations

BTS: blood transfusion services
CRF: case report form
CRP: C-reactive protein
CI: confidence interval
CTU: Clinical Trials Unit
DMC: Data Monitoring Committee
EDTA: ethylenediaminetetraacetic acid
ERC: Endpoint Review Committee
GCP: Good Clinical Practice
Hb: haemoglobin
HIV: Human Immunodeficiency Virus
HR: hazards ratio
ICH: International Committee on Harmonisation
MRC: Medical Research Council
MUAC: mid-upper arm circumference
MVMM: multi-vitamin multi-mineral
OR: odds ratio
RCT: randomised controlled trial
RNI: recommended nutritional intake
RUTF: ready-to-use therapeutic food
SA: severe anaemia
SAE: serious adverse events
SOC: standard of care
SOP: Standard Operating Procedure
SSA: sub-Saharan Africa
TMG: Trial Management Group
TRALI: Transfusion Related Acute Lung Injury
TSC: Trial Steering Committee
WHO: World Health Organisation

## References

[1] McLean E, Cogswell M, Egli I, Wojdyla D, de Benoist B. Worldwide prevalence of anaemia, WHO Vitamin and Mineral Nutrition Information System, 1993-2005. Public health nutrition. 2009;12(4):444-54. 10.1017/S1368980008002401 18498676

[2] World malaria report 2014. Geneva, Switzerland: World Health Organization, 2014

[3] Pocket book of hospital care for children. Guidelines for the management of common childhood illnesses. 2nd ed. Geneva: World Health Organization; 2013. 24006557

[4] Kiguli S, Maitland K, George EC, Olupot-Olupot P, Opoka RO, Engoru C (2015). Anaemia and blood transfusion in African children presenting to hospital with severe febrile illness. BMC Med.

[5] Brabin BJ, Premji Z, Verhoeff F (2001). An analysis of anemia and child mortality. J Nutr.

[6] Phiri KS, Calis JC, Faragher B, Nkhoma E, Ng'oma K, Mangochi B (2008). Long term outcome of severe anaemia in Malawian children. PLoS One.

[7] Calis JC, Phiri KS, Faragher EB, Brabin BJ, Bates I, Cuevas LE (2008). Severe anemia in Malawian children. N Engl J Med.

[8] Meremikwu M, Smith HJ (2000). Blood transfusion for treating malarial anaemia. Cochrane Database Syst Rev..

[9] Management of the child with a serious infection or severe malnutrition: guidelines for care at the first-referral level in developing countries. Geneva: WHO: World Health Organization, 2000.

[10] Holzer BR, Egger M, Teuscher T, Koch S, Mboya DM, Smith GD (1993). Childhood anemia in Africa: to transfuse or not transfuse?. Acta Trop.

[11] Bojang KA, Palmer A, Boele van Hensbroek M, Banya WA, Greenwood BM (1997). Management of severe malarial anaemia in Gambian children. Trans R Soc Trop Med Hyg.

[12] English M, Ahmed M, Ngando C, Berkley J, Ross A (2002). Blood transfusion for severe anaemia in children in a Kenyan hospital. Lancet.

[13] Lackritz EM, Campbell CC, Ruebush TK, Hightower AW, Wakube W, Steketee RW (1992). Effect of blood transfusion on survival among children in a Kenyan hospital. Lancet.

[14] Bojang KA, Van Hensbroek MB, Palmer A, Banya WA, Jaffar S, Greenwood BM (1997). Predictors of mortality in Gambian children with severe malaria anaemia. Ann Trop Paediatr.

[15] Ernest SK, Anunobi NE, Adeniyi A (2002). Correlates of emergency response interval and mortality from severe anaemia in childhood. West Afr J Med.

[16] Maitland K, Kiguli S, Opoka RO, Engoru C, Olupot-Olupot P, Akech SO (2011). Mortality after fluid bolus in African children with severe infection. N Engl J Med.

[17] Marsh K, Forster D, Waruiru C, Mwangi I, Winstanley M, Marsh V (1995). Indicators of life-threatening malaria in African children. N Engl J Med.

[18] Pedro R, Akech S, Maitland K (2010). Changing trends in blood transfusion in children and neonates admitted in Kilifi District Hospital, Kenya. Malar J..

[19] Hospital Care for Children: guidelines for the management of common illnesses with limited resources. Geneva, Switzerland: World Health Organization, 2005. ISBN 92 4 154670 0.

[20] Walker RH (1996). Mathematical calculations in transfusion medicine. Clin Lab Med.

[21] Abdalla SH (1990). Iron and folate status in Gambian children with malaria. Ann Trop Paediatr.

[22] van Hensbroek MB, Morris-Jones S, Meisner S, Jaffar S, Bayo L, Dackour R (1995). Iron, but not folic acid, combined with effective antimalarial therapy promotes haematological recovery in African children after acute falciparum malaria. Trans R Soc Trop Med Hyg.

[23] Sazawal S, Black RE, Ramsan M, Chwaya HM, Stoltzfus RJ, Dutta A (2006). Effects of routine prophylactic supplementation with iron and folic acid on admission to hospital and mortality in preschool children in a high malaria transmission setting: community-based, randomised, placebo-controlled trial. Lancet.

[24] Northrop-Clewes CA, Thurnham DI (2013). Biomarkers for the differentiation of anemia and their clinical usefulness. J Blood Med..

[25] Phiri KS, Calis JC, Siyasiya A, Bates I, Brabin B, van Hensbroek MB (2009). New cut-off values for ferritin and soluble transferrin receptor for the assessment of iron deficiency in children in a high infection pressure area. J Clin Pathol.

[26] Schauer C, Zlotkin S (2003). Home fortification with micronutrient sprinkles – A new approach for the prevention and treatment of nutritional anemias. Paediatrics Child Health.

[27] Christofides A, Schauer C, Sharieff W, Zlotkin SH (2005). Acceptability of micronutrient sprinkles: a new food-based approach for delivering iron to First Nations and Inuit children in Northern Canada. Chronic Dis Can.

[28] Chintu C, Bhat GJ, Walker AS, Mulenga V, Sinyinza F, Lishimpi K (2004). Co-trimoxazole as prophylaxis against opportunistic infections in HIV-infected Zambian children (CHAP): a double-blind randomised placebo-controlled trial. Lancet.

[29] Mulenga V, Ford D, Walker AS, Mwenya D, Mwansa J, Sinyinza F (2007). Effect of cotrimoxazole on causes of death, hospital admissions and antibiotic use in HIV-infected children. Aids.

[30] Walker AS, Ford D, Gilks CF, Munderi P, Ssali F, Reid A (2010). Daily co-trimoxazole prophylaxis in severely immunosuppressed HIV-infected adults in Africa started on combination antiretroviral therapy: an observational analysis of the DART cohort. Lancet.

[31] Mwenya DM, Charalambous BM, Phillips PP, Mwansa JC, Batt SL, Nunn AJ (2010). Impact of cotrimoxazole on carriage and antibiotic resistance of Streptococcus pneumoniae and Haemophilus influenzae in HIV-infected children in Zambia. Antimicrob Agents Chemother.

[32] Thera MA, Sehdev PS, Coulibaly D, Traore K, Garba MN, Cissoko Y (2005). Impact of trimethoprim-sulfamethoxazole prophylaxis on falciparum malaria infection and disease. J Infect Dis.

[33] Arinaitwe E, Gasasira A, Verret W, Homsy J, Wanzira H, Kakuru A (2012). The association between malnutrition and the incidence of malaria among young HIV-infected and -uninfected Ugandan children: a prospective study. Malar J..

[34] Adu-Afarwuah S, Lartey A, Brown KH, Zlotkin S, Briend A, Dewey KG (2007). Randomized comparison of 3 types of micronutrient supplements for home fortification of complementary foods in Ghana: effects on growth and motor development. Am J Clin Nutr.

[35] Christofides A, Asante KP, Schauer C, Sharieff W, Owusu-Agyei S, Zlotkin S (2006). Multi-micronutrient Sprinkles including a low dose of iron provided as microencapsulated ferrous fumarate improves haematologic indices in anaemic children: a randomized clinical trial. Matern Child Nutr.

[36] Lundeen E, Schueth T, Toktobaev N, Zlotkin S, Hyder SM, Houser R (2010). Daily use of Sprinkles micronutrient powder for 2 months reduces anemia among children 6 to 36 months of age in the Kyrgyz Republic: a cluster-randomized trial. Food Nutr Bull.

[37] Suchdev PS, De-Regil LM, Walleser S, Vist GE, Pena-Rosas JP. Multiple micronutrient powders for home (point of use) fortification of foods in pregnant women: a systematic review. Geneva: World Health Organization, 2011.

[38] Guidelines on co-trimoxazole prophylaxis for hiv-related infections among children, adolescents and adults. Geneva: World Health Organization, 2006.

[39] Maitland K, Molyneux S, Boga M, Kiguli S, Lang T (2011). Use of deferred consent for severely ill children in a multi-centre phase III trial. Trials..

[40] Molyneux S, Njue M, Boga M, Akello L, Olupot-Olupot P, Engoru C (2013). ‘The words will pass with the blowing wind’: staff and parent views of the deferred consent process, with prior assent, used in an emergency fluids trial in two African hospitals. PLoS One.

[41] Olupot-Olupot P, Engoru C, Thompson J, Nteziyaremye J, Chebet M, Ssenyondo T (2014). Phase II trial of standard versus increased transfusion volume in Ugandan children with acute severe anemia. BMC Med.

[42] WHO. Global database on blood safety: report 2004–2005. Geneva: 2008

[43] Tapko JB, Sam O, Diarra-Nama AJ (2007). Status of blood safety in the WHO African Region: report of the 2004 survey.

[44] Ala F, Allain JP, Bates I, Boukef K, Boulton F, Brandful J (2012). External financial aid to blood transfusion services in sub-Saharan Africa: a need for reflection. PLoS Med.

